# The Social Science of Institutional Transformation: Intersectional Change in the Academy

**DOI:** 10.3389/fsoc.2022.824497

**Published:** 2022-04-14

**Authors:** Shauna A. Morimoto

**Affiliations:** Department of Sociology and Criminology, University of Arkansas, Fayetteville, AR, United States

**Keywords:** intersectionality, academic institutions, social science, institutional transformation, STEM equity, knowledge

## Abstract

This article examines intersectional praxis as an approach to institutional transformation, arguing that intersectionality is both a catalyst for and outcome of gender equity efforts in the social sciences and other academic STEM fields. As such, approaching gender equity intersectionally can be understood as a way that theory and practice are co-constitutive in social science and hence an important aspect of transforming academic institutions. Through a case study of the US National Science Foundation (NSF) ADVANCE program for gender equity in STEM, I look at the development of ADVANCE from an effort to support women in scientific fields to becoming a program for institutional transformation grounded in an intersectional understanding of women's inequity in the academic labor force. I ask two related questions in the efforts to address gender inequities in STEM. First, what is the relationship between academic institutions (which are simultaneously sites for the discovery of knowledge and gender inequality) and the National Science foundation, as the premier American academic institutional funding agency? Second, how has this relationship, through those working on ADVANCE, fundamentally shifted the understanding of the social scientific tools and strategies necessary to advance equity for women in academia? In looking at these questions, I argue that, beyond women's representation in social sciences and academia broadly, intersectionality is an important scholarly advance in social science that offers a dialectical tool for change.

## Introduction

This article examines intersectional praxis as an approach to institutional transformation, arguing that intersectionality is both a catalyst for and outcome of gender equity efforts in the social sciences and other academic STEM fields. As such, approaching gender equity intersectionally can be understood as a way that theory and practice are co-constitutive in social science and hence an important aspect of transforming academic institutions. Through a case study of the US National Science Foundation (NSF) ADVANCE program for gender equity in STEM, I look at the development of ADVANCE from an effort to support women in scientific fields to becoming a program for institutional transformation grounded in an intersectional understanding of women's inequity in the academic labor force. I ask two related questions in the efforts to address gender inequities in STEM. First, what is the relationship between academic institutions (which are simultaneously sites for the discovery of knowledge and gender inequality), and the National Science foundation, as the premier American academic institutional funding agency? Second, how has this relationship, through those working on ADVANCE, fundamentally shifted the understanding of the social scientific tools and strategies necessary to advance equity for women in academia?

In answering these questions, I argue that, beyond women's representation in social sciences and academia broadly, intersectionality is an important scholarly advance that offers a dialectical tool for change. More than just a buzzword (Davis, 2008) an intersectional approach simultaneously calls attention to multiple sites and processes of institutional oppression and privilege while still being attentive to the individuals that occupy disadvantaged structural locations (Cho et al., 2013). For social scientists, therefore, intersectionality offers a praxis or practice that attends to structural inequality as well as the representation of individuals in addressing social change. Indeed, faculty who have taken on much of the work of institutional transformation are, themselves, also the targets of the structural reform that ADVANCE seeks to achieve. Social science fields including psychology, sociology, political science, anthropology and economics are classified as sciences by NSF definitions (Congressional Research Service, 2012). Social scientists involved in ADVANCE thus seek to solve inequities on a structural level, but also reflexively understand these issues as reflected in their own experiences (see Laube, 2021; McQuillan and Hernandez, 2021).

Because of this focus on gender representation, in most ADVANCE programs, gender is generally treated as binary, and equity efforts entail adopting programs for inclusivity and women's access to academic STEM fields. While there has long been attention to women's representation in the natural and physical sciences, women's access in the social sciences is also unequal. Economics and political science are also dominated by white men, with women representing 32% of political scientists and 24% of economists (Hur et al., 2017). While sociology and psychology have achieved overall gender parity (National Science Foundation [NSF], 2019), both fields have fewer women full professors (American Psychological Association, 2014; American Sociological Association, 2016) giving rise to concerns over a leaky pipeline (National Science Foundation [NSF], 2019). Moreover, BIPOC (black, indigenous, people of color) women are significantly underrepresented in academic sociology and psychology (American Sociological Association, 2016; Hur et al., 2017; Stewart and Vailan, 2018; National Science Foundation [NSF], 2019).

Adopting intersectionality as part of the ADVANCE program, therefore, had implications for the social sciences as STEM disciplines, as these fields developed strategies of structural change to address the ways that gender inequality is intersectionally defined, and in particular, the ongoing underrepresentation of BIPOC women in the academy (DeAro et al., 2019; Fox Tree and Vaid, 2022; Gregory, 2001). Through the ADVANCE program, intersectional approaches to inequality recognize the contributions of underrepresented women, while also calling upon the social sciences to devise institutionally based strategies to increase the representation of all women throughout the academy (Carbado et al., 2013).

Because strategies for gender equity are designed by each individual NSF ADVANCE institutional awardee, this case study of ADVANCE draws on the websites, proposals, reports and publications of a random sample of institutional transformation programs, examining the strategies adopted by these institutions. Looking at ADVANCE historically, I also consider the changes in the calls for ADVANCE proposals that guided these programs. I discuss the feedback loop among social scientists who are working toward gender equity, the funding agency and academic institutions in advancing intersectional change to facilitate women's representation.

Approaching gender equity intersectionally engages theory and practice as co-constitutive in the process of transforming academic institutions. This defines intersectionality interactively, or as the interplay between and among social actors and social institutions as they give meanings to categories such as “race,” “gender” and “class” (Ferree, 2009). Rather than something inherent in social structures, intersectionality emerges through a dynamic process that ensures that the role of social actors is not overlooked (Ferree, 2009). Intersectional analysis thus involves looking at the processes by which configurations of intersectional social relations and institutional sites arise (Choo and Ferree, 2010). By adopting intersectionality in programs to address equity in the academy, I argue that social sciences helped design strategies and inform notions of their own representation and overall mechanisms of institutional change (see Patton and Haynes, 2018).

I begin with a brief overview of NSF ADVANCE goals for gender equity through systemic change in academic STEM fields. I then discuss the evolution of the ADVANCE program in dialogue with institutional grantees, and the initiatives to address institutional inequity that the grantees implement. Next, I consider the explicit introduction of intersectionality into ADVANCE as an important discursive moment for fostering equity for women with intersectional identities, particularly BIPOC women. As social scientists adopted an intersectional lens, they furthered the possibilities of transformation through the intersectional production of knowledge and continue to move the academy to structural changes to generate a culture of equity through the recognition of minoritized women of color (Patton and Haynes, 2018).

## Case Study: NSF Advance

Gender equity in Science, Technology, Engineering and Mathematics (STEM) fields is a primary policy and higher education goal in the US and across many countries (Kodate et al., 2010; Smith, 2011; Morimoto and Zajicek, 2014; Rimmer and Sawer, 2016). The National Science Foundation (NSF) began the ADVANCE program as an effort to foster gender equity by facilitating STEM women faculty's access to and advancement in US academic institutions (DeAro et al., 2019). In the United States, gender equity is often couched in this binary, and specifically to ensure the talent and participation of the full workforce in order to maintain a leadership position in innovation and technology (Zippel and Ferree, 2017). As STEM fields are historically dominated by men, facilitating opportunities for women in STEM is critical to building the talent pool in technological fields and hence an important policy goal. The ADVANCE program was designed with the understanding that—for women to gain equity in the STEM workforce—they must also be teachers, mentors and leaders in scientific fields.

The US NSF ADVANCE program provides an important case study because the National Science Foundation is the primary funder of basic research and education in the social sciences in the US (Congressional Research Service., 2021) With one of NSF's primary goals being to “promote the progress of science,” this independent federal funding agency is governed by a director and a National Science Board that also serves in an advisory capacity to the US Congress and President (Congressional Research Service, 2012). Accordingly, the NSF's approach to creating equity in STEM fields influences and enables the how US universities understand and tackle this issue. Moreover, demands for greater inclusion informs policy—not just in the United States, but also in the United Kingdom and the European Union (Elomäki, 2015; Ferree and Zippel, 2015).

To achieve more inclusive STEM fields, in the early days of ADVANCE, research on gender equity came from studies that showed that organizations were inherently gendered and unequal (Ferguson, 1984; Acker, 1990). These concepts were applied to academia in an 1999 MIT report (MIT Report, 1999) that stated that gender inequity is embedded in the broad environment of academic culture and reinforced through micro-level institutional processes (National Academy of Sciences (US), 2006). Therefore, as programs for equity developed, efforts shifted from enabling individual women to successfully navigate academia and defining gender equity in terms of the number of women in academic positions, to finding ways to transform educational institutions into more equitable environments.

In the discussion that follows, this article considers how social scientific theory about gender equity developed alongside these programs for institutional change. Accordingly, social science STEM disciplines—particularly psychology and sociology—engaged in the practice of dismantling gender inequality within their institutions and disciplines through designing and implementing ADVANCE equity strategies. Accordingly, social scientists argued that transformation requires attending to intersectionality, or the complex ways that multiple axes of ability and constraint—including race, class, sexuality and physical ability, among others—limit women's access to academic careers and success (Browne and Misra, 2003; Ong, 2011; Wu and Jing, 2011; Morimoto and Zajicek, 2014; Armstrong and Jovanovich, 2017).

## Data and Methods

Because ADVANCE programs are designed and implemented by individual institutions, I conducted content analysis of ADVANCE documents from two randomly selected institutions in each of the ADVANCE Institutional Transformation cohorts 1-7 (2001-2014), and all of the social science projects in cohorts 8 and 9 (2016 and 2019), as these most recent cohorts represent the period for which NSF required intersectionality as an additional criteria in ADVANCE proposals. Project analysis included a review of all of the documents and websites associated with the NSF ADVANCE grant, including research proposals, reports, publications and white papers. This study included analysis of all of the social science supplemental projects, where available. Alongside the analysis of proposals, I reviewed the ADVANCE calls for proposals for the years from 2005 to 2016 (*n* = 5) to document changes in the call and conceptualization of the ADVANCE project (see also Laursen and De Welde, 2019).

Content analysis was performed on documents, deriving codes related to intersectionality and generating themes (Boyatzis, 1998). Initial categories were developed according to the intervention or social science phenomenon that was the subject of the study. Subsequent codes examined the ways that intersectionality was implicated or studied in the research, according to identities that modified gender such as URM, BIPOC or LGBTQ (Armstrong and Jovanovich, 2017), as well as the theory or social phenomenon that the social science project was engaging. Documents were then reexamined with codes in mind to understand what type of intersectional approach the projects were taking. Specifically, we noted whether intersectionality was treated as counting the number of women in various categories (i.e., BIPOC women, Latinas, etc.) and/or if intersectionality was emergent (i.e., social phenomena of inclusion or exclusion arose within organizational contexts, or if those contexts gave meanings to penalties and privileges). To the extent that a specific category of women were being studied, we noted this as well, along with the level of analysis of the ADVANCE project, and how or whether the study included structural change. Coding was conducted by the author and a research assistant, to allow for a check on the quality of coding and reconcile differences in document analysis.

In reviewing these documents, I sought to gain insight into how issues of gender equity were framed and what the theories or strategies social scientists relied on in seeking gender equity in academic institutions. In contextualizing the documentation in terms of the literature on ADVANCE and the projects coming out of ADVANCE, I seek to understand how changing concepts of addressing equity are reflected in the social scientific discovery that has come out of NSF ADVANCE. In addition, I assess the extent to which the evolution of the social science coming out of ADVANCE paves the way for women's representation and success in the academy and continues to impact how social scientists understand strategies to increase equity. It is important to note that the content analysis does not provide a rigorous overview or assessment of ADVANCE projects or their accomplishments and relies on data that is self-reported through project websites and materials. Moreover, I do not assess BIPOC women's outcomes quantitively. Instead, my goal is to understand how social science continues to evolve in seeking equity, arguing that an intersectional framework is an emergent and central component of change for the social scientists working on these grants, as well as for the disciplinary contexts in which they pursued institutional transformation.

## The First Generation of Advance: from a Program for Women to Institutional Transformation

At its inception, NSF ADVANCE offered competitive opportunities for women scientists to advance in academic institutions through fellowships, grant funding, and similar opportunities that targeted individual scientists (Armstrong and Jovanovich, 2017; DeAro et al., 2019). With an approach that focused on representation, early ADVANCE programming sought to provide funding for STEM women and thereby offer a path to their success. While this strategy allowed for an impact on a handful of women, it also implied that navigating the academy was an individual pursuit, and that, with assistance, women could and would become successful within the constraints of the institution. More pejoratively referred to as a strategy of “fixing women,” (Dalton, 2001; Stewart and Vailan, 2018) social science critics argued that the problems of inequity could not be resolved by supporting the careers of token experts, but instead the key to a more equitable scientific workforce entailed addressing the ways that academic institutions constrained and enabled faculty (Rosser and Lane, 2002; Rosser, 2017; DeAro et al., 2019).

Accordingly, to address inequality inherent in academic culture (MIT Report, 2010), ADVANCE also called for an institutional transformation (IT) track, which supported transforming the institutional contexts in which scientific and engineering knowledge is produced. Spurred by social science research indicating that institutional barriers can only be addressed by institutional-level solutions, the ADVANCE IT program was designed to effect change at the institutional level, rather than focusing on supporting careers of individual women (Rosser, 2006).

The decision to engage gender equity as a problem of transforming institutions derived from a long line of feminist thinking showing how gender—defined as a social relation, institution, and/or structure—is deeply embedded in the everyday operations of modern bureaucracies. Stemming from sociology, this scholarship showed the complexity of discriminatory structures, as well as the contradictory processes and the multiplicity of meanings and symbols permeating gendered organizations (Alvesson and Billing, 1992; Britton, 2000; Reskin, 2003; Ridgeway, 2009). Indeed, Acker's (1990) groundbreaking work on gendered organizations prompted the rapid development of scholarship on the organizational processes, practices, and mechanisms that create and reproduce gender inequalities. Consequently, feminist scholars replaced the notion that equity requires the abolition of bureaucracy (Firestone, 1970; Ferguson, 1984; Acker, 1990) with a sociological research that asserted that greater equity required the transformation of the bureaucratic institution (Britton, 2000; Britton and Logan, 2008; McQuillan and Hernandez, 2021). Thus, moving from an initial focus on women scientists and STEM disciplines, the ADVANCE IT program called for strategies to transform systematically the day-to-day operations of institutions of higher education in pursuit of gender equity (Rosser and Lane, 2002; DeAro et al., 2019) and ultimately, create a better workplace for all faculty (Stewart et al., 2007; Bilmoria and Liang, 2012; Laursen and Austin, 2020). Recognizing that structural barriers to gender equity are specific to institutional contexts, therefore, ADVANCE solicits grant proposals seeking to implement activities that will lead to greater gender equity in STEM fields by transforming those institutions.

Targeting these institutional barriers was thus borne from social science research, and a way for social scientists to address inequality in their own fields. As Valian and Stewart note, much of their ADVANCE work was informed by their experiences as academic psychologists (Stewart and Vailan, 2018; see also McQuillan and Hernandez, 2021). Accordingly, early ADVANCE grantees focused on social science research that corresponded with barriers to women's STEM equity such as lack of transparency and clarity in tenure and promotion and the absence of effective mentoring structures. For example, in the first IT cohort in 2001, Georgia Tech examined how gender affects mentoring and mentoring networks. Fox and Fonesca (2006) found that both women and men of higher rank are more likely to mentor, but men are more likely to mentor men only, while women are likely to mentor both men and women (Fox and Fonesca, 2006). Moreover, Fox and Fonesca (2006) show that mentoring is variable by institutional climate, but importantly, institutional climate varies by gender composition.

Also in an early cohort (2005), UNC Charlotte sought to address “the interplay between structural and social psychological factors that generate gender inequality” (University of North Carolina Charlotte, 2005). Through a number of initiatives aimed at recruitment, mentoring, leadership and salary equity, UNC Charlotte's ADVANCE team reported better climate and more women STEM faculty at the end of their granting period. At the same time, however, the number of underrepresented minority faculty declined during this time (Lorden et al., 2013). Consistent with NSF proposal requirements of this cohort, the initial Charlotte project used a social science framing that addressed gender inequity, and included analysis of underrepresented minority (URM) faculty. Importantly, their approach centered on identifying and solving inequities through institutional research rather than developing an underlying understanding of the mechanisms that created that inequality (Devine et al., 2017; Laursen and Austin, 2020).

In developing attention to multiple sites of inequality, ADVANCE social science researchers at University of Nebraska-Lincoln (cohort 2008) examined the networks of faculty in STEM to understand how faculty were connected and what these connections meant for long-term faculty success. Networks analysis provided insight into individual and departmental connections and isolation, as well as access to collaborative, mentorship and social networks for faculty members, finding that women and non-white faculty are more likely to be peripheral network actors (Falci, 2009; Falci and Watanabe, 2020).

Over time, the National Science Foundation became increasingly explicit about the social science aspect of the projects, and the use of social science theory and methods to investigate persistent inequalities (Laursen and De Welde, 2019). In addition to articulating the planned activities for structural equity within institutions, starting in 2010, NSF called for a research project to accompany the main activities of the grant, indicating “IT projects must include a 5-page research component designed to study the effectiveness of the proposed innovations in order to contribute to the knowledge base informing academic institutional transformation” (National Science Foundation [NSF], 2010). In doing so, NSF incorporated social science into the ADVANCE program for institutional transformation.

These criteria became more explicit in their connection to social science research, when, in 2014, the solicitation was revised to indicate: “the supplemental document must include information relevant to the proposed study, such as: (1) the disciplinary and conceptual framework for the project; (2) a discussion of the theory or theories grounding the research and the testable hypotheses; (3) the proposed methods to test the hypotheses; (4) the expected findings; and (5) to what extent the results and data will be disaggregated for multiple characteristics such as race, ethnicity, sexual orientation and disability, in addition to gender” (National Science Foundation [NSF], 2014). In addition to requiring a disciplinary framework for the research project associated with the proposed grant supplement, the project had to include a theory of change, a testable hypothesis and, in foreshadowing the move toward intersectionality, the extent to which the project would address “multiple characteristics…in addition to gender” (National Science Foundation [NSF], 2014).

In an example of how institutions adapted to the changing requirements of the solicitation, Montana State examined barriers to women's careers and structured their ADVANCE project on self-determination theory, which is rooted in psychology and holds that meeting the psychological needs of autonomy, relatedness and competence provide motivation and lead to success (Deci and Ryan, 2012). Montana State structured the interventions at their institution to address these needs by focusing on building women's research capacity, creating supportive interactions and relationships and integrating work-life balance. Their projects showed improvements for women faculty and increased hiring of women on campus. The social science project supported self-determination theory as improving inclusivity on campus and garnering more participation for women in STEM (Smith, 2012).

Despite these successes, with ADVANCE projects primarily focused on theoretical frameworks to address gender equity, early ADVANCE projects were criticized for implicitly or de facto targeting and thus benefiting white women (Hunt et al., 2012; Armstrong and Jovanovich, 2017; Fox Tree and Vaid, 2022). Indeed, studies showed that while white women were beginning to make equity gains in academic STEM fields, women of color lagged behind (Hirshfield and Tiffany, 2012; McQuillan and Hernandez, 2021), particularly black women (Snyder et al., 2016; Buchanan, 2020; Fox Tree and Vaid, 2022). In the social sciences, this is quite noteworthy, with less racial and ethnic diversity in these fields than in men dominated fields of engineering and biomedicine (Hur et al., 2017).

Attentive to these issues, however, through their reliance on self-determination theory, programs like Montana State learned that structurally addressing the inequality of (white) women lead to improved outcomes for faculty of color and other marginalized faculty members. Likewise, Oregon State relied on systems oppression theory with the goal of “disrupting systems of oppression,” addressing inequality intersectionally by encouraging administrators and faculty to develop a “critical consciousness” that would generate more inclusive interpersonal interactions and a more positive atmosphere. Importantly, the researchers at Oregon State argued that a critical consciousness is particularly important at predominantly white institutions, hence adding an intersectional element to shifting the climate in the study of structural gender inequality.

As these earlier projects demonstrated, even without an explicit call for intersectional research or an intersectional framework, intersectional concerns emerged in research that seeks to address structural inequalities. Indeed, an intersectional framework coincides with the multiple goals of ADVANCE to address systemic inequality while also being attentive to individuals that occupy locations of opportunity and constraint. With an approach that entails addressing empowerment of those at the margins through community engagement, social critique, coalition building and establishing resistance (Rosenthal, 2016) intersectionality also crosses social scientific disciplinary boundaries by considering both the individual and their context as paramount to changing outcomes and social transformation. Accordingly, intersectional concerns emerged in ADVANCE projects because they reflected reality. Such reality is consistent with intersectionality as the ways that the on-going renegotiation and reconceptualization of individual identities exposed how “systems of inequality grant or prohibit access to power” (Warner et al., 2018a, p. 526).

## The Second Generation of Advance: Gender Equity and Intersectionality

Thus, supported by findings at ADVANCE schools such as Oregon State and Montana State, social scientists argued noted that gender inequity could not be addressed independent of addressing other penalties and privileges associated with identity (Hunt et al., 2012; Armstrong and Jovanovich, 2017; DeAro et al., 2019). Accordingly, in 2016, NSF revised the ADVANCE solicitation to indicate that intersectionality was an additional merit review criteria for addressing gender inequality in academic STEM fields and all proposals for ADVANCE grants were required to conceptualize their projects accordingly (National Science Foundation [NSF], 2016; DeAro et al., 2019).

With its roots in black feminist thought that was critical of second wave (white) feminism as exclusively concerned with the plight of white women (Davis, 1981; Lourde, 1984; Crenshaw, 1989) intersectionality as a framework for ADVANCE projects resonated with what some ADVANCE scholars were already advocating by showing that that oppression is linked—or intersects—on axes of race, class, gender, sexuality and other sites of social hierarchy (King, 1988; Crenshaw, 1989). Intersectional theorists examined this “matrix of domination” (Collins, 1990), showing ways that systems of oppression “mutually construct one another” (Collins, 1998), while social scientists began unpacking how to operationalize and apply intersectionality in efforts for social change (McCall, 2005; Ferree, 2009; Cho et al., 2013).

The National Science Foundation's requirement of an intersectional component importantly signaled the NSF's endorsement of this orientation as pivotal to questions of equity. In doing so, intersectionality, as a critical concept, also became central to the way ADVANCE social science scholarship approached change and equity. With a focus on the academy, ADVANCE scholars revealed the complexity and the many dimensions of inequity in academic intersectional practices, policies, and authority structures, through the development of an intersectional approach to transformation of academic institutions. Illuminating how gender inequities were not independent of or simply additive to other barriers to success in the academy, research shows that BIPOC women are chronically underrepresented in academia generally, and particularly in STEM fields (Li and Koedel, 2017; National Science Foundation [NSF], 2019). Indeed, psychology has the highest proportion of White faculty of the social sciences (Fox Tree and Vaid, 2022).

The change in the ADVANCE program to include intersectionality in the call for proposals was therefore arguably inevitable because multiple dimensions of inequality emerged when ADVANCE programs addressed “gender only” equity in STEM. Moreover, scholars critiqued sublimating non-white identities in intersectional projects and thus voiced support for the revision of the ADVANCE solicitation guidelines to include a call for intersectionality (Hunt et al., 2012; Armstrong and Jovanovich, 2017). Doing so resulted in the development of a social science coming out of ADVANCE that was more multidimensional, allowing for more expansive insight into the workings of subtle power relationships in the day-to-day operations of academic institutions. In operationalizing intersectionality, therefore, ADVANCE scholars focused on “how things work, rather than who people are” (Cho et al., 2013; Warner et al., 2018b). Accordingly, in addition to giving voice to the marginalized, incorporating intersectionality into ADVANCE ensured attention was paid to how those in the dominant group access power (Warner et al., 2018a, p. 527). ADVANCE projects and related research thus identified social science phenomena such as cognitive and implicit biases, bystander impact and intervention, cumulative disadvantage and microaggressions as significant factors in gender inequality. Further, by focusing on equity in STEM, NSF ADVANCE became a locus for intersectional thinking among non-social science STEM fields (see Nielsen et al., 2017, 2018).

Florida International University (FIU), for example, implemented a project on bystander awareness, as a behavioral intervention aiming to make faculty more appreciative of diversity and less likely to harbor prejudicial attitudes as part of their early ADVANCE funding. FIU also sought to increase the affirmation of diversity by teaching the social skills necessary to intervene when confronted with bias and discrimination (Florida International University, 2021). Combining this approach with their social science project on microclimates and developing a network of other institutions in Florida, FIU's project explicitly targeted broad issues of diversity, equity and inclusion as a way to understand and address gender inequity intersectionally. By considering climate issues and educating and empowering all faculty about their role in creating more diverse and inclusive environments, FIU's program focuses on social phenomena that arise in microclimates and contribute to inequality on multiple levels.

Similarly, UMass Lowell addressed microaggressions in their social science project. This project sought to gain insight into how microaggressions constrained all faculty, with a particular focus on how faculty of color experience gendered microaggressions and the attitudes that study participants developed toward microaggressions. In addition, the UMass Lowell team sought to understand how identity (for both majority and underrepresented groups) affects barriers to intervening in microaggressions. Thus, at both UMass Lowell and FIU, social scientists examined the responsibility of both dominate and marginalized groups in bringing about social change.

In other recent ADVANCE cohorts, institutions seek to understand how inequities are embedded and emergent in the structure and development of the academy and academic careers. For example, Arizona State University takes a life course perspective in examining the structure of pathways to leadership at an interdisciplinary institution. Their approach allows them to see how gender, race, ethnicity, foreign-born status, sexual orientation and disability shape faculty career pathways and leadership opportunities. Approaching the problem holistically and structurally allows the researchers to highlight how categorical markers of inequality constrain and enable faculty throughout their scientific careers.

UMass Amherst ADVANCE, conversely, takes an approach that emphasizes on-going data collection and development of plans and progress across the institution. The ADVANCE team then leverages these largescale data to structure and inform change targeting the “relationships, resources and recognition” that create and promote successful faculty members. With both a baseline climate survey and the continual collection and examination of institutional data, the ADVANCE team is able to understand how faculty access to resources and development of inclusive communities emerge based on gender, race, sexuality, nationality, rank and other factors. Data collection such as this points the ADVANCE team in directions to help them understand problems as they emerge—and hence enabled the team to address, for example, the COVID crisis and its intersectional impact (University of Massachusetts Amherst, 2021). By examining data from the ground up, this ADVANCE team can see how intersectional concerns structurally emerge and tackle those concerns alongside their planned areas of intervention.

## Discussion and Conclusions: Operationalizing Intersectionality and Institutional Transformation

By operating at multiple levels of analysis, therefore, intersectionality addresses the complexity of both the barriers to equity and the ways to address inequity. Feminist scholars have long held that reflexivity about knowledge-intensive institutions and academic institutions in particular, is critical because—so long as academic institutions remain inequitable—the project of science and discovery of knowledge remain hegemonically masculine (Harding, 1986, 1991), white (Collins, 1990) and heterosexist (Foucault, 1978). Although the ideology of some academic disciplines, such as engineering, is more tightly coupled with the image of disembodied white heterosexual hegemonic masculinity (Bix, 2004; Leonard and Nicholls, 2013), the image of a scientist, scientific excellence, and hegemonic masculinity undergirds the broader organization of science and the discovery of knowledge (Harding, 1986, 1991, 2008; Ong, 2005; Allison, 2007; Wilcox, 2009).

Importantly, STEM researchers, particularly but not exclusively, those in sociology and psychology social sciences, became instrumental in designing and implementing the strategies to seek gender equity in their fields. Both from their own experiences of bias in the academy and drawing on findings of inequalities within institutions and organizations, social scientists—as STEM researchers, practitioners and women navigating their own academic careers—became key players in strategies for dismantling gender bias in academic settings (see McQuillan and Hernandez, 2021).

When putting ADVANCE projects with an intersectional component into practice, social science scholars demonstrate that IT is not an abstraction, but also intimately tied to *real embodied workers*. Thus, equating organizational success—both practically and symbolically—with real, embodied workers rather than a disembodied ideal can generate more equitable organizational practices in the academy as well as other organizations. Importantly, since transforming organizations involves being attentive to the ebb and flow of cross-constitutive organizational structures and practices (Holvino, 2010). Interactive intersectionality asks us to continually and actively be on guard to the ways that inequalities arise and must be addressed. Moreover, it forces us to continually consider the context and assumptions that give rise to those inequalities (Ferree, 2009; Choo and Ferree, 2010; Cho et al., 2013).

According to Ferree, “it is an empirical matter in any given context to see what concepts are important to the configuration of inequalities in discourse and in practices by people in many different social positions, and locational studies of intersectionality can contribute to this discovery process” (Ferree, 2009, p. 89). Therefore, by operationalizing intersectionality with the understanding that the meanings of gender, race, class and any number of other social categories are produced and reinforced in and through social organization, we can see how confronting and dismantling these structures in the academy necessarily leads to new knowledge and experiences.

As a theory or framework for action, therefore, intersectionality is less precise than other models of change. Concepts with clearer and more measurable outcomes are likely to be counted as more successful in garnering calculable progress (Britton, 2010; Springer, 2020). In particular, representation is the easiest way to identify success; if more women are in working and getting promoted in US academic institutions, then the NSF ADVANCE program is working (Bilmoria and Liang, 2012; McQuillan and Hernandez, 2021). Likewise, if more BIPOC women are in STEM, then including a call for intersectionality as a requirement for ADVANCE programs is also a success. As Nelson and Zippel (2021) point out, social science theoretical concepts that can be demonstrated and provide measurable remedies for change are likely to gain high traction in addressing inequalities, particularly if those inequalities are intersectionally located.

Yet intersectionality, in and of itself, is not measurable in such a clear way. Nor does it guarantee a quick—or perhaps even long term—change or turnaround in representation of BIPOC women on university faculties. But easy measurement, particularly of representation, has its limitations as well. As Ray (2019) explains, for change, we have to continually look at the organizational context and changes within those structures to see shifts. With this in mind, and as the expansion of the research on institutional change continues to make progress, current research looks toward institutional transformation as a process involving non-predominantly white institutions (PWI), since the majority of ADVANCE grants have gone to PWIs (Bird and Kowalski, 2022), which necessarily inhibits the ability to change either representation or organizational structure. Further, questions about equity and inclusion force a rethinking about inclusionary and exclusionary categories, as women-as-binary approaches exclude women identifying and trans faculty (t philosopher, 2019).

The success of social sciences to bring about change is harder to quantify but easier to see in the shifts in institutional culture (McQuillan and Hernandez, 2021). Laube (2021) finds that feminist sociologists have the field of vision and analytic tools to work toward institutional transformation, and the ability to adapt and expand those tools to continually promote change. Likewise, Settles et al. (2020) argue for structural changes in the field of psychology that allow for epistemic inclusion of intersectional scholarship and scholars. Embedding of practices and concepts that enable equity is also important to creating change, and likely more enduring (McQuillan and Hernandez, 2021). Moreover, when equity practices become “the way we do things around here” those practices are less likely to encounter resistance and concern (Bird and Lattimer, 2019).

Adopting intersectionality as a cornerstone of the ADVANCE program is part of the praxis of such a cultural shift in academic institutions. The NSF ADVANCE IT program began with the premise that the production of knowledge is rooted in an inequitable organizational structure. In conjunction with the funding agency, award grantees and social science discovery, the inception of an intersectional framework entails the production of knowledge that allows for academic institutions as a space for resistance and an opportunity for transformation. Accordingly, as an intersectional stance becomes part of the everyday business of equity within academics, it both facilitates equity efforts in the academy, and is a cultural outcome of those efforts. While academic institutions often seem stuck in maintaining conventional paths to institutional power, an intersectional approach to equity forces a rethinking of social science knowledge. And it is through the diversity of knowledge that comes with intersectionality that new knowledge is most likely to emerge (Patton and Haynes, 2018; Laursen and De Welde, 2019; Hofstra et al., 2020).

## Author Contributions

The author confirms being the sole contributor of this work and has approved it for publication.

## Funding

This article was supported in part by a grant from the National Science Foundation, NSF Adaptation Grant #2017744. Publication fees were supported by research funds from the University of Arkansas.

## Conflict of Interest

The author declares that the research was conducted in the absence of any commercial or financial relationships that could be construed as a potential conflict of interest.

## Publisher's Note

All claims expressed in this article are solely those of the authors and do not necessarily represent those of their affiliated organizations, or those of the publisher, the editors and the reviewers. Any product that may be evaluated in this article, or claim that may be made by its manufacturer, is not guaranteed or endorsed by the publisher.
